# Molecular typing of PPRV strains detected during an outbreak in sheep and goats in south-eastern Gabon in 2011

**DOI:** 10.1186/1743-422X-10-82

**Published:** 2013-03-11

**Authors:** Gael D Maganga, Delphine Verrier, Rodrigo M Zerbinati, Christian Drosten, Jan F Drexler, Eric M Leroy

**Affiliations:** 1 Centre International de Recherches Médicales de Franceville, Franceville, Gabon; 2 Institute of Virology, University of Bonn Medical Centre, Bonn, Germany; 3 Institut de Recherche pour le Développement, UMR MIVEGEC (IRD 224/CNRS5290/UM1/UM2), Montpellier, France

**Keywords:** Peste des petits ruminants virus, Molecular typing, Sheep, Gabon

## Abstract

**Background:**

Peste des petits ruminanats (PPR) is an economically important viral disease affecting goats and sheep. Four genetically distinct lineages of peste des petits ruminants virus (PPRV) have been identified. In Gabon, the virus has not so far been detected.

**Findings:**

Epidemiological investigations of Aboumi PPR outbreak revealed a high case fatality rate in sheep (98.9%). We detected and characterized peste des petits ruminants virus (PPRV), in October 2011, during the suspected outbreak in sheep and goats in Aboumi village located in the south-eastern. PPRV RNA was detected in 10 of 14 samples from three sick animals. Phylogenetic analysis revealed that the PPRV strain belonged to lineage IV and was closely related to strain circulating in neighboring Cameroon.

**Conclusions:**

This is the first molecular detection and typing of the PPRV strain associated with fatal PPR infection in these small ruminants and concrete evidence that PPRV is present and circulating in Gabon.

## Introduction

Peste des petits ruminants (PPR) is a highly contagious disease caused by PPR virus (PPRV, family *Paramyxoviridae*, genus *Morbillivirus)*. The genome of PPRV encodes for eight proteins: the nucleocapsid protein (N), the phosphoprotein (P), the matrix protein (M), the fusion protein (F), the haemagglutinin protein (H), the polymerase protein (L) and the two non-structural proteins, C and V. Interaction of the PPRV H and F proteins with the host plasma membrane leads to viral entry by binding of the H protein to receptors (signal lymphocyte activating molecules and other unidentified receptors). Briefly, the P protein regulates transcription and replication and assembly of the N protein to nucleocapsids, the M proteins mediate viral assembly. The role of C and V proteins in PPRV is still not clear [1].

PPR primarily affects sheep and goats, with case fatality rates reaching 90% in naive populations [2]. Animal affected by PPR shed the virus in exhaled air, in secretions and excretions (from the mouth, eye and nose, and in feces, semen, and urine) approximately 10 days after the onset of fever. PPR is one of the main transboundary animal diseases and represents a major threat to small ruminants [3]. It is considered the most economically important viral disease of sheep and goats in enzootic regions, where it has a major impact on the food supply.

**Figure 1:**
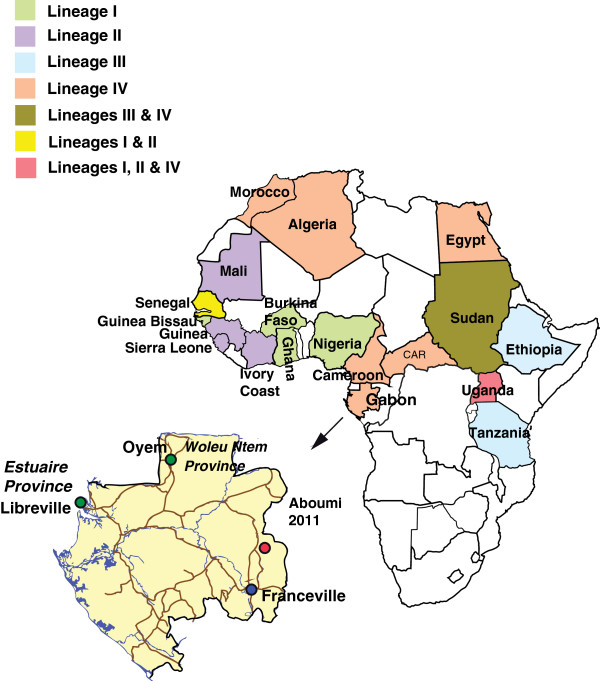
**Distribution of PPRV in Africa, based on molecular typing, and expanded map of Gabon.** Green circles show areas with serological evidence of PPRV circulation. Red circle indicate the location of PPRV-RNA-positive outbreak. The blue circle indicates the location of CIRMF.

Serological and clinical data suggest that PPRV is widely distributed across Africa, the Arabian Peninsula, the Middle East and Asia [4,5]. Four genetically distinct lineages of PPRV have been identified, three of which (I, II, III) were first described in Africa (Figure 1), including Guinea, Ivory Coast, Senagal, Mali, Burkina Faso, Ghana, Nigeria, Uganda and Tanzania, and the fourth (IV) in Asia. However, the Asian lineage was recently introduced in some African countries, including Cameroon and Central African Republic [3], Sudan and Morocco [6], Egypt [7], Algeria [8] and Uganda [9] (Figure 1).

In Gabon (Central Africa), PPR has previously been reported in 1993 on the basis of clinical and/or serological evidence [3,10], but the virus has not so far been detected. Since outbreaks have occurred sporadically. One of the largest outbreaks was recorded in June 1996 [10].

In this report we describe an outbreak of PPR in goats and sheep in Gabon and provide the first molecular characterization of the PPRV lineage associated with fatal PPR infection in these small ruminants.

## Materials and methods

Approval was given by the Gabonese Ministry of Agriculture, Livestock, Fisheries and Rural Development (Direction Générale du Développement Rural - Authorization N°0041/MAEPDR/SG/DGDR/DRE) to carry out the sampling and the diagnosis of the causative agent of the disease observed in small ruminants in ABOUMI outbreak. The samples were taken following the recommendations in the OIE Terrestrial Manual.

### Epidemiological investigations

In October 2011, at the request of local authorities, we investigated a disease outbreak that was occurring among domestic sheep and goats in Aboumi village, south-eastern Gabon (Figure 1). On October 7, animals with clinical signs of PPR were examined by a field veterinarian. Animal owners and villagers were interviewed to determine the history of disease in this area.

### Sample collection

Nine clinical samples, including sera as well as oral, nasal and ocular swabs, were collected from three sick animals. Five additional samples (liver, spleen, lung, intestine and mediastinal lymph node) were collected post-mortem from one animal that died within 1 hour after clinical sampling (Table 1). Samples were then carried on ice to the Centre International de Recherches Médicales de Franceville (CIRMF, Gabon) for analysis.

**Table 1:** Number and types of animal samples and results of reverse transcription PCR

**Species**	**Animal identifier**	**Samples**	**No. of samples collected and tested**	**No. (%) PPRV-positive**
Sheep	Aboumi 1	Ocular and nasal swabs, liver, lung, spleen, intestine, mediastinal lymph node tissue, serum	8	6 (75)
	Aboumi 2	Nasal swab, serum	2	1 (50)
	Aboumi 3	Ocular, oral and nasal swabs, serum	4	3 (75)

### Diagnosis

Swabs were suspended in 500 μl of 0.9% saline solution and total RNA was extracted from 100 μl of supernatant of sample on a BioRobot EZ1 automat (Qiagen) using the EZ1 Virus Mini Kit version 2.0 in the presence of DNase (Qiagen) according to manufacturer’s instructions [11]. RNA was used for molecular analysis.

Samples were first tested with a hemi-nested reverse transcription-polymerase chain reaction (hnRT-PCR) for the genus *Morbillivirus*, using generic primers targeting the polymerase gene [12]. All negative samples were retested with a specific real-time reverse transcription–PCR assay developed in-house as part of our study, using primers/probe lying within the amplified fragment (see Additional file 1 for details).

### Molecular characterization

Reverse transcription PCR (RT-PCR) using NP3/NP4 N genes primers’, targeting the N protein gene was carried out in combination with nucleotide sequencing as previously described [13]. The nucleic acid sequences obtained from PCR products were aligned with PPRV sequences retrieved from GenBank. Multiple aligments of the N gene were performed by using the ClustalW algorithm of the Mega 4 software package [14]. Phylogenetic analysis based on the 255 nucleotides of the N gene was done [6] with MrBayes V.3.2 software, using the default chain for two million generations with the GTR+G+I nucleotide substitution model [15]. Trees were sampled every 100 generations, resulting in 20 000 saved trees, the first 5000 saved trees being discarded as burn-in.

## Results

### Clinical and epidemiological findings

The outbreak occurred from September 28 to October 14, when 91 (98.9%) of 92 sheep and 2 (18.2%) of 11 goats died within a few days. No extension of the outbreak to neighboring villages was observed. Epidemiological investigations revealed that no mass mortality of small ruminants had previously been reported in Aboumi or in neighboring villages 8 to 13 km away. We found three sheep displaying signs of PPR, including fever (>40°C), purulent ocular and nasal discharge, diarrhea and respiratory distress.

### Detection of PPRV RNA

PPRV was identified, by hnRT-PCR in only one clinical sample from the three sick animals. In contrast, PPRV RNA was detected by the specific real-time RT-PCR assay in 10 of 14 samples from the three sick animals, which displayed elevated viral loads (cycle threshold range: 15–23). Samples positive for PPRV included swabs (oral, nasal and ocular) and liver, lung, spleen and intestine tissue. All sera tested by the the specific real-time RT-PCR assay were negative.

### Phylogenetic analysis

**Figure 2:**
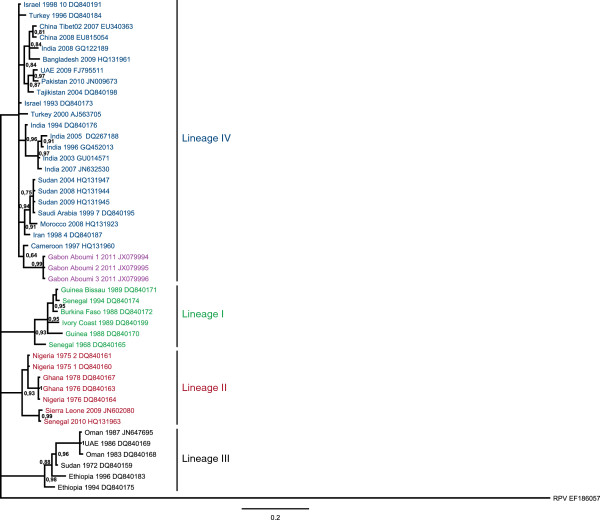
**Phylogenetic relationship between PPRV detected in Aboumi in 2011 and PPRV sequences found in GenBank.** The tree is based on 255 nucleotides of the nucleocapside (N) protein gene. The tree was visualized with FigTree 1.3.1. Bayesian posterior probabilities values are shown to the left of the branches; values below 0.75 were removed for clarity, except for the posterior probability value from the Cameroon-Gabon cluster. PPRV sequences obtained in this study are shown in purple.

Partials N gene sequences of 303 bp located on the C terminus end of nucleoprotein of virus were obtained. The 10 nucleotide sequences from the three sick animals were identical. Three nucleotide sequences (Gabon Aboumi 1, Gabon Aboumi 2 and Gabon Aboumi 3), each from a different animal, were aligned with PPRV sequences available in Genbank, and were themselves submitted to GenBank (Accession numbers JX079994 – JX079996). Phylogenetic analysis revealed that the Aboumi outbreak strain clustered in lineage IV, along with Asian strains (Figure 2). Moreover, this strain was closely related (95% nucleotide sequence identity) to the Cameroonian strain, with which it formed a distinct cluster (Figure 2).

## Discussion

In the present study, we describe an outbreak of PPR in goats and sheep in south-eastern Gabon. The virus was particularly virulent in sheep. While sheep mortality and serological evidence of PPRV infection were reported in the Estuaire and Woleu-Ntem provinces (North) between 1993 and 1996 [10], our findings provide the first concrete evidence (through the viral RNA detection) that PPRV is present and circulating in Gabon.

The hemi-nested reverse transcription-PCR targeting the polymerase gene is a broader Morbillivirus PCR using consensus degenerate primers. It was used as the first screening test because it was the only molecular test available to us to make the diagnosis. In general, degenerate primers can cause a drop in sensitivity of a test. PPRV has been detected in one clinical sample with the hemi-nested RT-PCR. This result was probably due to first differences in amount of viral RNA in different samples and second to drop in sensitivity cause by degenerate primers [12]. In contrast, real-time RT-PCR uses specific primers and probe lying within the partial sequence of PPRV obtained. Real-time RT-PCR is more sensitive and specific than hemi-nested RT-PCR. Hence, the real-time RT-PCR allowed the detection of PPRV RNA in a larger number of samples than hemi-nested RT-PCR.

Phylogenetic analysis revealed that the strain involved in the Aboumi outbreak belonged to lineage IV. This is compatible with the recent establishment and spread of the Asian PPRV lineage IV in Africa and its emergence in Central Africa [6]. The Gabonese PPRV strain clustered with the Cameroon strain. Further analysis of this cluster suggested that the PPRV strains in Gabon and Cameroon evolved separately from a common ancestor introduced in Central Africa.

The origin of the PPRV strain involved in the Aboumi outbreak is unclear. However, we suspect initial introduction of the Asian lineage IV in neighboring Cameroon and its spread to Gabon through importation of living animals. Many inhabitants of northern Gabon (Woleu-Ntem province) have families in southern Cameroon, thus faciliting animal trade and smuggling. In addition, animal movements are usually uncontrolled at the border. PPRV lineage IV has previously been identified in goats in the semi-arid northern area of Cameroon, but it was also found in all departments of southwestern Cameroon [16], near Woleu-Ntem province, where PPR infections have been described. Furthermore, Aboumi village is located at about 25 km from the border with the Republic of Congo (RC), where serological evidence of PPRV has been reported [3]. Although the lineage IV has not been characterized in the RC yet, we cannot exclude an introduction of the PPRV strain involved in the Aboumi outbreak from this latter country. The recent extensive PPRV outbreak reported in the DRC in June 2012, which killed more than 75,000 goats [17], could indeed provide another potential route of virus spread to Gabon via the RC.

In conclusion, we report the first molecular detection and typing of a PPRV strain involved in an outbreak with a high case fatality rate in Aboumi village, Gabon, in October 2011. The Gabonese strain was genetically closely related to the strain identified in neighboring Cameroon, where PPRV is actively circulating. These findings stress the importance of controls on animal movements, particularly in rural areas. A PPR monitoring program is needed to identify the strains circulating in Gabon and vaccine strategy should be implemented in endemic areas of Gabon.

## Competing interests

The authors declare that they have no competing interests.

## Authors’ contributions

MGD conducted sample collection, PCR experiments, phylogenetic analysis and wrote the manuscript. VD participated in sample collection organization and wrote the manuscript. ZRM did PCR experiments. DC and DJF designed real-time reverse transcription–PCR assay. LEM organized sample collection, wrote the manuscript and provided final approval of the manuscript. All authors read and approved the final manuscript.

## Supplementary Material

Additional file 1 Real-time RT-PCR protocol. Click here for file
