# Supplementary material for: Molecular typing of PPRV strains detected during an outbreak in sheep and goats in south-eastern Gabon in 2011
Source: Virol J. 2013 Mar 11;10:82. doi: 10.1186/1743-422X-10-82 (PMC3599724; doi:10.1186/1743-422X-10-82)
Supplement: Additional file 1 — Real-time RT-PCR protocol. [file 1743-422X-10-82-S1.pdf]

Additional file 1

Real-time RT-PCR protocol

Twenty-five microliter reactions used the TaqMan Universal PCR Master Mix (Applied Biosystems). Reaction contained 10µM of the respective primers (PPRV-GB-rtF, 5’-GAAGAATGAGTGGGATTCGGTTT-3’ and PPRV-GB-rtR, 5’-CGCCTTGACTCTGTCCCTCTA-3’) and probe (PPRV-GB-rtP, FAM-CCCGAAAGAATACCTCCGTTACAACCCA-BHQ1), 12.5µl Master Mix (Applied Biosystems), 1 µg bovine serum albumin (BSA), and 5 µl cDNA previously synthesized using the High Capacity cDNA Reverse Transcription Kit (Applied Biosystems, USA). The amplification cycle involved 2 min at 55°C and an initial denaturation at 95°C for 10 min, followed by 45 cycles of 95°C for 15 seconds, and 58°C for 1 min. Fluorescence was measured at the 58°C annealing/extension step.
